# CXCR1 and CXCR2 enhances human melanoma tumourigenesis, growth and invasion

**DOI:** 10.1038/sj.bjc.6605055

**Published:** 2009-04-28

**Authors:** S Singh, K C Nannuru, A Sadanandam, M L Varney, R K Singh

**Affiliations:** 1 Department of Pathology and Microbiology, University of Nebraska Medical Center, Omaha, NE, USA

**Keywords:** CXCL-8, CXCR1, CXCR2, human melanoma, tumourigenesis, invasion

## Abstract

The aggressiveness of malignant melanoma is associated with differential expression of CXCL-8 and its receptors, CXCR1 and CXCR2. However, the precise functional role of these receptors in melanoma progression remains unclear. In this study, we investigate the precise functional role of CXCR1 and CXCR2 in melanoma progression. CXCR1 or CXCR2 were stably overexpressed in human melanoma cell lines, SBC-2 (non-tumourigenic) and A375P (low-tumourigenic) exhibiting low endogenous expression of receptors. Functional assays were performed to study the resulting changes in cell proliferation, motility and invasion, and *in vivo* tumour growth using a mouse xenograft model. Our data demonstrated that CXCR1- or CXCR2-overexpressing SBC-2 and A375P melanoma cells had enhanced proliferation, chemotaxis and invasiveness *in vitro*. Interestingly, CXCR1 or CXCR2 overexpression in SBC-2 cells induced tumourigenicity, and A375P cells significantly enhanced tumour growth as examined *in vivo*. Immunohistochemical analyses showed significantly increased tumour cell proliferation and microvessel density and reduced apoptosis in tumours generated from CXCR1- or CXCR2-overexpressing melanoma cells. CXCR1- or CXCR2-induced modulation of melanoma cell proliferation and migration was observed to be mediated through the activation of ERK1/2 phosphorylation. Together, these studies demonstrate that CXCR1 and CXCR2 play essential role in growth, survival, motility and invasion of human melanoma.

Melanoma is the most common skin cancer associated with significant mortality (Lee, 1992). According to an estimate by American Cancer Society, 62 480 new cases of melanoma will be diagnosed in 2008 and 8420 people will die due to this disease in the United States (Jemal *et al*, 2008). Although early disease can be treated by surgery and a minor benefit noted with adjuvant therapy, no effective treatment is available for the advanced disease (Herlyn, 2006; Lee, 1992, 1996). Hence, an in-depth understanding of the biology of melanoma progression is highly required.

CXCL-8, a member of the CXC chemokine family, has been reported to induce migration, stimulate angiogenesis and promote tumour cell growth in melanoma and other malignancies (Matsushima and Oppenheim, 1989; Wang *et al*, 1990; Koch *et al*, 1992; Schadendorf *et al*, 1993; Youngs *et al*, 1997; Miyamoto *et al*, 1998; Bar-Eli, 1999; Singh and Varney, 2000; Li *et al*, 2002). CXCL-8 is a multifunctional cytokine/growth factor that binds to two high-affinity receptors: type A CXCL-8 receptor (IL-8RA/IL-8RI or CXCR1) and type B CXCL-8 receptor (IL-8RB/IL-8RII or CXCR2; Holmes *et al*, 1991; Murphy and Tiffany, 1991). Although CXCR2 binds with high affinity to CXCL-8 and other CXC chemokines such as CXCL-6, CXCL-5, CXCL-7 and CXCL-1, CXCR1 is less promiscuous and binds only to CXCL-8 (Matsushima and Oppenheim, 1989; Oppenheim *et al*, 1991; Miller and Krangel, 1992). Both these receptors have been implicated in the migration of neutrophils, monocytes and lymphocytes (Holmes *et al*, 1991; Murphy and Tiffany, 1991; Peiper *et al*, 1995; Moser and Loetscher, 2001). CXCR1 and CXCR2 share a high degree of sequence similarity (75.8% in amino-acid sequence), but differ within the extracellular and intracellular loops and the NH2- and COOH-terminal domains (Jones *et al*, 1995).

The expression of CXCL-8 in melanoma has been shown to correlate positively with disease progression (Singh *et al*, 1994; Ugurel *et al*, 2001). The aggressiveness of malignant melanoma is attributed, in part, to the expression of CXCL-8 and its receptors, CXCR1 and CXCR2 (Varney *et al*, 2006). In addition to melanoma cells, both CXCR1 and CXCR2 are differentially expressed on endothelial cells (Clark-Lewis *et al*, 1993; Addison *et al*, 2000; Li *et al*, 2002, 2003; Varney *et al*, 2003). Several studies have implicated CXCR1 and CXCR2 as important players in tumour progression (Brat *et al*, 2005; Murphy *et al*, 2005; Kline *et al*, 2007). We have also shown that neutralisation of CXCR1 or CXCR2 affects cell proliferation and migration, indicating the involvement of these receptors in altered cellular responses (Varney *et al*, 2003). However, the functional significance of CXCR1 and CXCR2 and its ligand CXCL-8 in melanoma progression remains unclear.

In this study, we hypothesised that CXCR1 and CXCR2 regulate melanoma tumour growth and progression. We have performed *in vitro* and *in vivo* functional assays to provide direct evidence for multiple overlapping roles of CXCR1 and CXCR2 in melanoma growth, tumourigenesis, motility and invasion in two cell line models (SBC-2, non-tumourigenic and A375P, low-tumourigenic).

## Materials and methods

### Cell lines, culture conditions and stable transfection

The human melanoma SBC-2 (non-tumourigenic) and A375P (low-tumourigenic) cell lines were cultured in RPMI-1640 or Dulbecco's Modified Eagle Medium (MediaTech, Herndon, VA, USA) at 37°C with 5% CO_2_ in humidified atmosphere. Culture media were supplemented with 5% fetal bovine serum (FBS; MediaTech), 1% L-glutamine (MediaTech), 1% vitamin solution (MediaTech) and gentamycin (Invitrogen, Carlsbad, CA, USA). Plasmids (pcDNA 3.1/Neo) carrying CXCR1 or CXCR2 cDNA were obtained from the University of Missouri cDNA Resource Center, Rolla, MO, USA. The insert sequence and orientation were confirmed by sequencing the clones. SBC-2 and A375P cells (5 × 10^5^ cells per dish) were seeded in 100 mm culture dishes (Falcon, Lincoln Park, NJ, USA) in complete medium. Twenty-four hours later (at 60–80% confluence), the cells were transfected with pcDNA3.1/Neo (SBC-2-control or A375P-control), pcDNA3.1/Neo-CXCR1 (SBC-2-CXCR1 or A375P-CXCR1) or pcDNA3.1/Neo-CXCR2 (SBC-2-CXCR2 or A375P-CXCR2) plasmids using Lipofectamine (Invitrogen) following the manufacturers protocol. Forty-eight hours later, the cells were switched to a selective medium containing Geneticin (G418; 400–800 *μ*g ml^−1^; Invitrogen). Resistant pooled populations were selected and maintained in medium supplemented with G418.

### RNA isolation and RT–PCR analysis

Total RNA was isolated using Trizol reagent (Invitrogen). To confirm the ectopic expression of CXCR1 and CXCR2 mRNA, we performed reverse transcriptase-based PCR (RT–PCR) analysis, using vector-specific (forward) and receptor-specific (reverse) primers. Briefly, cDNA was synthesised from 5 *μ*g total RNA using SuperScript II Reverse Transcriptase (Invitrogen) and oligo(dT) primer. Two microlitres of first strand cDNA (1 : 10 dilution) was amplified using the following primers: pcDNA 3.1/Neo, 5′-CTAGCGTTTAAACTTAAGCTTGGTA-3′ (forward), CXCR1, 5′-AGGGCAGGGACAGATTCATAG-3′ (reverse) and CXCR2, 5′-GGGCCAGGAGCAAGGACAGA-3′ (reverse); control GAPDH, 5′-ACGCATTTGGTCGTATTGGG-3′ (forward) and GAPDH, 5′-TGATTTTGGAGGGATCTCGC-3′ (reverse). Amplified products were resolved through a 1.5% agarose gel containing ethidium bromide and analysed using an Alpha Imager gel documentation system (AlphaInnotech, San Leandro, CA, USA).

### Western blot analysis

Cells were processed for protein extraction and western blotting using standard procedures. Briefly, the cells were washed twice with PBS and scraped in Triton X-100 buffer (1% Triton X-100, 50 mmol l^−1^ TBS (pH 7.4), 10 mmol l^−1^ EDTA with protease inhibitors (Roche Diagnostics, Mannheim, Germany) and phosphatase inhibitors (5 mM NaF and 5 mM Na_3_VO_4_; Sigma Chemicals, St Louis, MO, USA)). Cell lysates were passed through the needle syringe to facilitate the disruption of the cell membranes and were centrifuged at 14 000 r.p.m. for 20 min at 4°C, and supernatant were collected. The proteins (50 *μ*g) were resolved by electrophoresis on 8% SDS–PAGE and transferred onto a polyvinylidene difluoride membrane. Subsequently, the membranes were subjected to standard immunodetection procedure using specific antibodies: anti-CXCR1 and anti-CXCR2 (1 : 50, mouse monoclonal; R&D Systems, Minneapolis, MN, USA); anti-GAPDH (1 : 1000, rabbit monoclonal; Cell Signaling Technology, Beverly, MA, USA); pERK1/2 and ERK1/2 (1 : 1000, rabbit polyclonal; Cell Signaling Technology). Secondary horseradish peroxidase-conjugated antibodies (Santa Cruz Biotechnology, Santa Cruz, CA, USA) were used at 1 : 2000 dilution. To examine ERK1/2 mitogen-activated protein kinase (MAPK) signalling, cells were treated for 1 h with 20 *μ*M PD98059 (Calbiochem, Gibbstown, NJ, USA), a specific inhibitor of ERK1/2 MAP kinase. All the blots were processed with ECL Plus Western Blotting detection kit (GE Healthcare, Piscataway, NJ, USA), and the signal was detected by a Typhoon 9410 Variable Mode Imager.

### *In vitro* cell growth assay

Cells were seeded in 96-well plates at low density (5000 cells per well (SBC-2-transfected); 1000 cells per well (A375P-transfected)). Following overnight adherence, cells were incubated with media alone or medium containing different serum concentrations, with or without CXCL-8 (10 ng ml^−1^) for 72 h. Cell proliferation was determined by MTT (3-(4,5-dimethylthiazol-2-yl)-2,5-diphenyltetrazolium bromide, a tetrazole) assay as previously described (Singh and Varney, 1998; Li *et al*, 2001). Growth increase was calculated as percent (%)=[{(*A*/*B*)−1} × 100], where *A* and *B* are the absorbance of treated (CXCL-8-stimulated) and untreated cells (media alone), respectively.

### Cell motility and invasion assay

To investigate the effect of overexpression of CXCR1 or CXCR2 on cell migration, cells (1 × 10^6^ cells per well) in serum-free media were plated in the top chamber of non-coated polyethylene terephthalate membranes (six-well insert; 8 *μ*m pore size; Becton Dickinson, Franklin Lakes, NJ, USA). For invasion, cells (10 000 cells per well) were plated onto Matrigel-coated transwell chambers (24-well insert; 8 *μ*m pore size; Corning Costar Corp., Cambridge, MA, USA) in serum-free media. The bottom chamber contained 1.0 ml serum-free media with or without CXCL-8 (10 ng ml^−1^). The cells were incubated for 24 h at 37°C and cells that did not pass through the membrane pores were removed. Migrated cells were stained using Hema 3 kit (Fisher Scientific Company L.L.C., Kalamazoo, MI, USA) as per the manufacturer's instructions and counted in 10 random fields (200 × ). To study the role of MAPK signalling in cell migration, transfected SBC-2 and A375P cells were treated with 20 *μ*M PD98059 (Calbiochem MAPK) inhibitor for 1 h. Cells were than analysed for migration.

### Tumour growth analysis

To test the tumourigenic capacity and growth kinetics, group of 5 nude mice (*n*=5) were injected subcutaneously (s.c.) in the dorsal flank with 3 × 10^6^ cells (SBC-2-CXCR1, SBC-2-CXCR2 or SBC-2-control) or 1 × 10^6^ cells (A375P-CXCR1, A375P-CXCR2 or A375P-control). Tumour volume was measured twice weekly with a calliper and was calculated by using the formula *π*/6 × (smaller diameter)^2^ × (larger diameter). Mice were killed on day 45 (A375P-group) and on day 75 (SBC-2-group) after tumour implantation. All studies were done in accordance with the Institutional Animal Use and Care Committee of the University of Nebraska Medical Center. For histological examinations, tissues were fixed in zinc fixative and embedded in paraffin.

### Immunohistochemistry

Immunohistochemical analysis was performed as previously described (Varney *et al*, 2006) using the following antibodies: mouse monoclonal anti-proliferating cell nuclear antigen (PCNA; 1 : 40; Santa Cruz Biotechnology), mouse monoclonal anti-CXCR1 (1 : 100; R&D systems), mouse monoclonal anti-CXCR2 (1 : 50; R&D systems) and mouse biotinylated GS-IB4 (isolectin from *Griffonia simplicifolia*; 1 : 50; Vector Laboratories, Burlingame, CA, USA). Tumour cell apoptosis was measured using terminal deoxyribonucleotidyl transferase dUTP nick end labelling (TUNEL). Briefly, tumour sections were deparaffinised by incubation in EZ-Dewax (Biogenex, San Ramon, CA, USA) and rinsed in distilled water to remove residual EZ-Dewax. Following non-specific blocking for 30 min, sections were incubated with primary antibodies overnight at 4°C. Sections were then washed and subsequently incubated at room temperature with the respective biotinylated secondary antibodies (1 : 500 in PBS) for 45 min, except for GS-IB4. Immunoreactivity was visualised by incubation with avidin–biotin complex and diaminobenzidine tetrahydrochloride substrate (Vector Laboratories). The sections were observed and stained cells and vessels were counted microscopically (Nikon, Melville, NY, USA) using 5 × 5 reticle grid (Klarmann Rulings, Litchfield, NH, USA).

### Statistical analysis

All values are expressed as mean±s.e.m. Differences between the groups were compared using the unpaired two-tailed *t*-test in SPSS software (SPSS Inc., Chicago, IL, USA). *In vivo* analysis was performed using Mann–Whitney *U*-test for significance. A *P* value of equal or less than 0.05 was considered statistically significant.

## Results

### Isolation and characterisation of clones overexpressing CXCR1 or CXCR2

**Figure 1:**
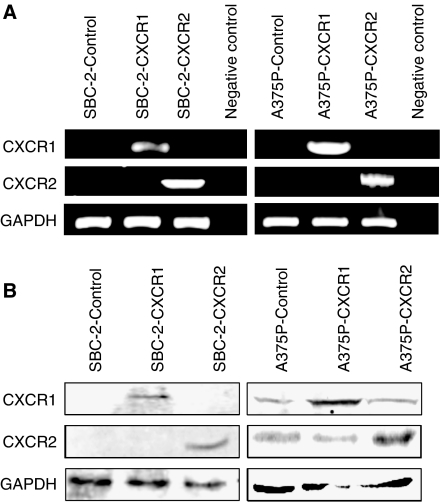
CXCR1 and CXCR2 expression in SBC-2 and A375P melanoma cells. Cells were stably transfected with vector control, or with vector containing CXCR1 or CXCR2 cDNA insert. (**A**) RT–PCR analysis shows increased expression of CXCR1 or CXCR2 mRNA in selected pooled clones. GAPDH was used as a control. (**B**) Western blotting showing increased expression of CXCR1 and CXCR2 in overexpressing melanoma cells as compared with control cells. GAPDH was used as a loading control. This is a representative gel picture of at least three experiments with similar results.

To test the functional role of CXCR1 and CXCR2 in human melanoma, SBC-2 and A375P melanoma cells were stably transfected with empty vector (pcDNA 3.1/Neo) or vector containing CXCR1 or CXCR2 cDNA. G418-resistant clones derived from the control vector, CXCR1- or CXCR2-transfected cells were isolated, expanded and the stable overexpression of CXCR1 and CXCR2 was determined in the pooled sublines by RT–PCR (Figure 1A) and western blot (Figure 1B). The stable overexpression of CXCR1 or CXCR2 in SBC2 and A375P cells compared with vector control was also monitored by immunocytochemical analyses (Supplementary Figure 1A). Receptor expression was observed on both the cell surface and cytoplasm. Results of immunocytochemical analyses were consistent with the western blot analysis, further confirming the stable overexpression of CXCR1 or CXCR2.

### Overexpression of CXCR1 or CXCR2 enhanced tumour growth

**Figure 2:**
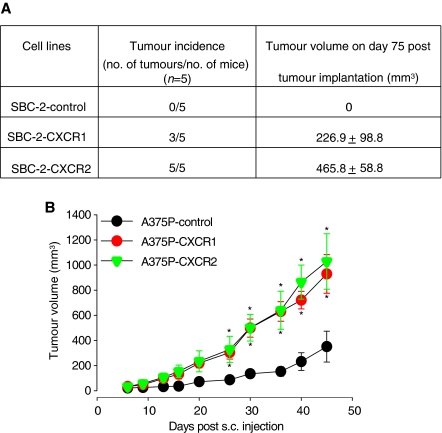
CXCR1 and CXCR2 expression enhances melanoma tumour growth. Stably transfected SBC-2 (3 × 10^6^ cells in 0.1 ml of HBSS) or A375P (1 × 10^6^ cells in 0.1 ml of HBSS) cells were subcutaneously (s.c.) injected into the flank of nude mice. In SBC-2-group, tumour incidence represents the number of tumours formed per number of injected mice and tumour volume on day 75 after tumour injection (**A**). For A375P-group tumour volume from day 0 to day 45 (*n*=5) (**B**). Growth of A375P-CXCR1 and A375P-CXCR2 increased significantly compared with A375P-control. Results are shown as mean±s.e.m. ^*^Significantly different from controls (*P*<0.05).

To determine the role of CXCR1 or CXCR2 in melanoma tumour growth, SBC2-transfected (SBC-2-CXCR1, SBC2-CXCR2 or SBC-2-control) and A375P-transfected (A375P-CXCR1, A375P-CXCR2 or A375P-control) cells were injected subcutaneously in nude mice and monitored for tumour growth. Mice injected with SBC-2-CXCR1 or SBC2-CXCR2 cell lines showed a palpable tumour within 30 days after injection, whereas the mice injected with A375P-CXCR1, A375P-CXCR2 or A375P-control cell lines showed a tumour within a week. The mice injected with SBC-2-control did not show any tumour formation as expected (Singh *et al*, 1995). Mice injected with SBC-2-CXCR1 cells, had 60% tumour incidence, whereas the mice with SBC-2-CXCR2 had 100% tumour incidence (Figure 2A). In the A375P group (A375P-CXCR1, A375P-CXCR2 or A375P-control), all the mice developed tumours. SBC-2-CXCR1 cells injected s.c. produced tumours with an average volume of 226.9±98.8 mm^3^, whereas, SBC-2-CXCR2 tumours had an average volume of 465.8±58.8 mm^3^. Additionally, mice injected with A375P-CXCR1 or A375P-CXCR2 cells exhibited significantly enhanced tumour growth (2.6 to 2.9-fold, *P*<0.05) as compared to A375P-control cells-injected mice (Figure 2B). Sustained expression of CXCR1 and CXCR2 in tumour tissues was confirmed by immunohistochemical staining (data not shown).

### Increased proliferation and decreased apoptosis in CXCR1 or CXCR2 expressing melanoma tumours

**Figure 3:**
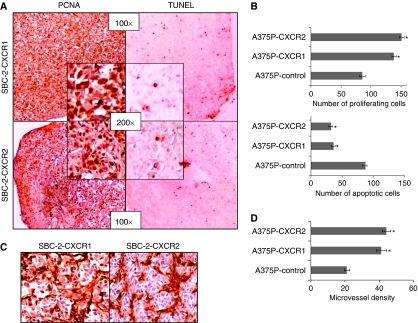
CXCR1 and CXCR2 expression enhances *in vivo* melanoma cell proliferation, survival and tumour neovascularisation. Immunohistochemical staining for PCNA and TUNEL were analysed based on DAB staining as described in Materials and Methods. In SBC-2-group, tumours showed an imbalance of PCNA to TUNEL-positive cells (**A**). For A375P-group PCNA (**B**, upper panel) and TUNEL (**B**, lower panel) positive cells were counted in 10 arbitrarily selected fields at 200 × magnification in a double-blinded manner and expressed as average number of cells per field view±s.e.m. Immunohistochemical staining for microvessel using anti-GS-IB4 was analysed as described in Materials and Methods. Tumours from SBC-2 group were highly vascular (**C**). For A375P tumours, quantification of microvessel density at 200 × magnification in 10 random fields was examined. The values are average number of immunostained positive cells±s.e.m. (**D**). ^*^Significantly different from control (*P*<0.05).

As induced and enhanced tumour growth may be the result of increasing proliferation or decreasing apoptosis, we examined the contribution of these two processes by PCNA and TUNEL immunostaining of tumours. As SBC-2-control did not form tumours, no comparison in number of PCNA and TUNEL-positive cells was done with SBC-2-CXCR1 or SBC-2-CXCR2 tumours. Interestingly, a higher number of PCNA-positive compared to TUNEL-positive cells was observed in SBC-2-CXCR1 and SBC-2-CXCR2 tumours (Figure 3A). The average number of PCNA-positive cells for A375P-CXCR1 (1.6-fold; *P*<0.05) and A375P-CXCR2 (1.8-fold; *P*<0.05) were significantly higher as compared to A375P-control tumours (Figure 3B, upper panel; Supplementary Figure 2A). Similarly, the average number of TUNEL-positive cells in A375P-CXCR1 (36%) and A375P-CXCR2 (32%) were significantly lower as compared to A375P-control tumours (87%), demonstrating a 2.4-fold (A375P-CXCR1) and 2.7-fold (A375P-CXCR2) decrease compared to control tumours (Figure 3B, lower panel; Supplementary Figure 2B).

### Overexpression of CXCR1 or CXCR2 enhanced tumour neovascularisation

Most solid tumours, including melanoma, require new blood vessel formation to grow beyond a few millimetres in diameter (Folkman, 1995). Having examined the effect of overexpression of CXCR1 or CXCR2 on tumourigenicity, we next examined their roles in facilitating neovascularisation *in vivo*. The number of blood vessels was compared in tumour tissues by immunohistochemical staining using biotinylated isolectin B4 (GS-IB4). The average number of blood vessels was counted, excluding necrotic areas. The tumours from SBC2-CXCR1 and SBC-2-CXCR2 were highly vascular (Figure 3C). We observed a 2.0-fold (A375P-CXCR1) and a 2.3-fold (A375P-CXCR2) increase in the number of tumour blood vessels as compared to the A375P-control tumours (Figure 3D).

### CXCR1 or CXCR2 enhanced human melanoma cell growth, motility and invasiveness *in vitro*

**Figure 4:**
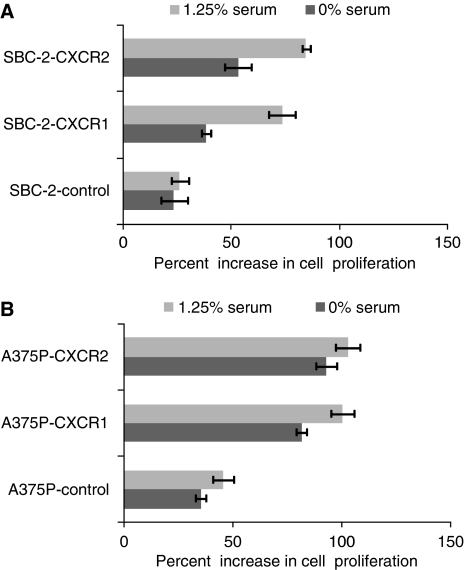
Overexpression of CXCR1 and CXCR2 affect *in vitro* melanoma cell growth. SBC-2 (5000 cells per well) (**A**) and A375P cells (1000 cells per well) (**B**) in a 96-well plate were cultured in media with (1.25%) or without serum. Cellular growth was determined at 72 h by MTT assay. Growth increase was calculated as percent (%)=[{(*A*/*B*)–1} × 100], where *A* and *B* are the absorbance of treated (CXCL-8 stimulated) and untreated cells (media alone), respectively. The values are mean percent inhibition of growth±s.e.m.

**Figure 5:**
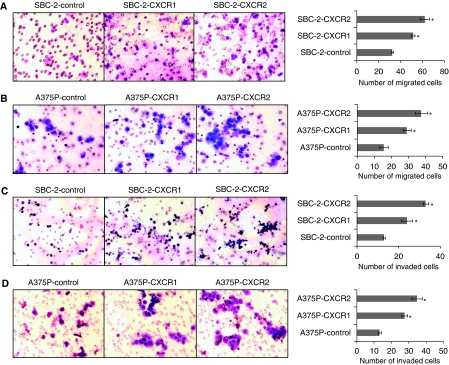
CXCR1 and CXCR2 overexpression enhances cell motility and invasion. SBC-2 and A375P cells expressing CXCR1 or CXCR2 were seeded on non-coated or Matrigel-coated membranes for motility (**A** and **B**) and invasion (**C** and **D**) assays, respectively, and incubated for 24 h. Serum-free medium containing 10 ng ml^−1^ CXCL-8 was added to the lower chamber. The cells that did not migrate through the Matrigel and/or pores in the membrane were removed, and cells on the other side of the membrane were stained and photographed at 200 × magnification. Cells were counted in 10 random fields (200 × ) and expressed as the average number of cells per field of view. The values are number of migrated cells±s.e.m. This is a representative of three experiments done in triplicate. ^*^Significantly different from control CXCL-8-treated cells (*P*<0.05).

Having seen the observable differences in tumourigenicity, we next performed *in vitro* assays to examine the effect of CXCR1 and CXCR2 overexpression on the growth and malignant behaviour of melanoma cells. Tumour cell growth was examined by MTT assay. Both the cell line models (SBC-2 and A375P) resulted in a progressive increase in growth *in vitro* assays (Figure 4A and B). Our data also demonstrated a significant (*P*<0.05) increase in CXCL-8-induced chemotaxis of SBC-2-CXCR1 (1.6-fold) or SBC-2-CXCR2 (1.9-fold) cells as compared to the SBC-2-control cells (Figure 5A). A375P-CXCR1 and A375P-CXCR2 cells also showed a significant (*P*<0.05) increase in the number of migrating cells by 2.2- 2.9-fold as compared with A375P-control in an *in vitro* migration assay (Figure 5B). Next, we examined whether the increase in cell motility was also associated with the enhanced invasive potential of these cells. Our results showed that the number of invading cells in SBC-2-cells was 1.8-fold (SBC-2-CXCR1) and 2.5-fold (SBC-2-CXCR2) higher as compared to SBC-2-control cells (Figure 5C). Similarly, A375P cells were 2.1-fold (A375P-CXCR1) and 2.7-fold (A375P-CXCR2) higher as compared to the A375P-control cells (Figure 5D).

### ERK1/2 mitogen-activated protein kinase pathway is involved in CXCR1- or CXCR2-mediated melanoma cell growth and motility

**Figure 6:**
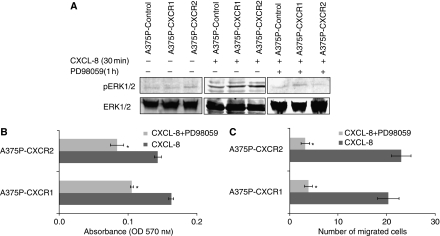
CXCR1 and CXCR2 activate ERK1/2 phosphorylation and mediated melanoma cell growth and motility. (**A**) For ERK phosphorylation, A375P cells overexpressing CXCR1 or CXCR2 were stimulated with CXCL-8 (10 ng ml^−1^) for 0 or 30 min (left and middle panels). Effect of ERK1/2 inhibitor was examined by treating the cells for 1 h and stimulated with CXCL-8 for 30 min (right panel). Cells were lysed, and equal amounts of protein were analysed by western blot analysis using antibodies against pERK1/2 and ERK1/2 antibody. Bound immunocomplexes were detected using ECL Plus chemiluminescence detection reagent. (**B**) For Growth, A375P-transfected cells were seeded (1000 cells per well) in 96-well plates and incubated with serum-free medium containing CXCL-8 (10 ng ml^−1^) with or without PD98059 (20 *μ*M). Cellular growth was determined at 72 h by MTT assay. The values are average absorbance±s.e.m. (**C**) A375P-transfected cells treated for 1 h with 20 *μ*M PD98059 were used for migration assay as described in Materials and Methods. Migrated cells were counted in 10 random fields (200 × ). The values are number of migrated cells±s.e.m. This is a representative of three experiments done in triplicate. ^*^Significantly different from control cells (*P*<0.05).

The MAP kinase pathway is important for growth, differentiation, survival and migration (Dunn *et al*, 2005; Yoon and Seger, 2006). CXCL-8 has already been shown to induce intracellular signalling pathways activated by CXCR1 and CXCR2 by phosphorylation of ERK1/2 MAP kinase (Venkatakrishnan *et al*, 2000; Prado *et al*, 2007). To explain the mechanism by which the CXCL-8-CXCR1/2 axis is involved in the growth and migration of melanoma cells, we investigated the involvement of ERK1/2 MAP kinases. The treatment of A375P (A375P-CXCR1 and A375P-CXCR2) cells with CXCL-8 (10 ng ml^−1^) induced ERK1/2 phosphorylation more potently in comparison with the control cells and was maximal by 30 min (Figure 6A, left and middle panel). Furthermore, treatment of the cells with PD98059 completely suppressed the activation of ERK in the presence of CXCL-8 (Figure 6A, right panel). Similarly, SBC-2 cells (SBC-2-CXCR1, SBC-2-CXCR2 or SBC-2-control) treated with CXCL-8 (10 ng ml^−1^) induced ERK1/2 phosphorylation and PD98059 treatment suppressed the activation (data not shown).

To specifically assess the role of the ERK1/2 MAP kinase pathway in growth, cells were treated with 20 *μ*M PD98059±CXCL-8 in serum-free medium and growth was determined by MTT assay. Our results clearly showed a decrease in cell growth of CXCR1 or CXCR2 overexpressing A375P melanoma cells on treatment with the inhibitor (Figure 6B). Similarly, pretreatment of cells with PD98059 also significantly reduced CXCL-8-induced migration (Figure 6C). Similar results were observed in SBC-2-transfected cells (data not shown) Together, these results demonstrate that overexpression of CXCR1 or CXCR2 in SBC-2 and A375P melanoma cells leads to CXCL-8-induced activation of ERK1/2 MAP kinase, which facilitates increased cell growth and motility.

## Discussion

CXCR1 and CXCR2 are overexpressed on melanoma cells (Li *et al*, 2002, 2003; Varney *et al*, 2003), but their precise functional role in human melanoma progression is not well established. In this study, we demonstrated the functional significance of CXCR1 and CXCR2 expression in melanoma growth, tumourigenesis, motility and invasion. Our data showed that either of the receptors provided a growth advantage to melanoma cells and regulated melanoma tumourigenesis and growth by increased cell survival. Moreover, an increase in chemotaxis and chemoinvasion of human melanoma cells expressing CXCR1 or CXCR2 on stimulation with CXCL-8 was also observed. In addition, our data demonstrated that the CXCL-8-induced and CXCR1- or CXCR2-dependent modulation of melanoma cell proliferation and migration was mediated through the ERK1/2 MAP kinase pathway.

It is of great interest that the overexpression of CXCR1 or CXCR2 in SBC-2, a non-tumourigenic cell line induced tumour formation. Our previous studies have demonstrated that upregulation of CXCL-8 expression by ultraviolet rays or ectopic expression of CXCL-8 enhanced tumourigenesis in SBC-2 cells (Singh *et al*, 1995). Although the tumour incidence was identical when A375P-transfected cells were injected in nude mice, the tumours in A375P-CXCR1- and A375P-CXCR2-injected mice grew twice as larger than the control-injected mice. Therefore, the fact that tumour growth was induced and enhanced by overexpression of CXCR1 or CXCR2 in melanoma cells suggests the importance of CXCR1 and CXCR2 as key determinants of melanoma tumourigenesis and growth. Additional support is provided by the observation that tumours in SBC-2 (SBC-2-CXCR1 and SBC-2-CXCR2) and A375P (A375P-CXCR1 and A375P-CXCR2) groups had increased tumour cell proliferation and survival, further suggesting the importance of CXCR1 and CXCR2 in the regulation of phenotypes associated with melanoma growth. These results strengthen our previous report that modulation of CXCL-8 expression in melanoma cells enhances tumour growth and metastasis (Singh *et al*, 1994; Singh and Varney, 1998).

Our previous results have demonstrated that CXCL-8 is a paracrine and autocrine angiogenic factor (Li *et al*, 2003, 2005) and CXCL-8-mediated angiogenesis is an important step in melanoma growth. We hypothesised that this process might be augmented in both groups. Our results show that tumour vascularisation was increased in SBC-2 (SBC-2-CXCR1 and SBC-2-CXCR2) and A375P (A375P-CXCR1 and A375P-CXCR2) groups of mice compared with their respective control mice. The observed increase in neovascularisation was similar to enhanced tumourigenesis and cell proliferation *in vivo*. Hence, once again our data emphasise the importance of CXCR1 and CXCR2 expression in melanoma cells.

Interestingly, our data also demonstrated a greater invasive potential of SBC-2 and A375P cells expressing CXCR1 or CXCR2 as compared to control cells. Differential roles of CXCR1 and CXCR2 in endothelial cell migration have been reported previously (Li *et al*, 2002, 2003). It was shown that the migration of CXCR1- and CXCR2-expressing endothelial cells is predominantly mediated through CXCR2 (Addison *et al*, 2000; Loukinova *et al*, 2000; Schraufstatter *et al*, 2001; Heidemann *et al*, 2003). On the other hand, a recent report suggests that in melanoma cells, CXCL-8-mediated chemotaxis is mainly mediated through CXCR1 (Ramjeesingh *et al*, 2003). We, however, did not observe any significant differences in CXCL-8-dependent motility and invasion between CXCR1- or CXCR2-overexpressing cells. This is consistent with our previous observation, where we showed that neutralisation of either CXCR1 or CXCR2 *in vitro* inhibits microvascular endothelial cell migration (Li *et al*, 2003, 2005). Cumulatively, our findings support that overall growth of the tumour depends on several factors including the rate of tumour cell proliferation, survival and vascularisation of the tumour tissue along with the invasive capacity of the tumour cells (Jain *et al*, 1998; O’Hayre *et al*, 2008).

Here, we have also demonstrated that overexpression of CXCR1 or CXCR2 in CXCL-8-expressing melanoma cells potentiates ERK1/2 MAP kinase signalling. However, we did not observe a difference in ERK1/2 phosphorylation between melanoma cells expressing CXCR1 or CXCR2, which further suggests the involvement of both receptors in the MAPK pathway. Furthermore, our study demonstrated that when the melanoma cells were incubated with an ERK1/2 inhibitor, CXCL-8-enhanced proliferation and migration was decreased. The MAP kinase signalling pathway is important for controlling various cellular functions such as growth and migration (Widmann *et al*, 1999; Kampen *et al*, 2000). The ERK1/2 MAP kinase pathway is constitutively activated in most melanoma cells, where it plays a major role in mediating their survival and proliferation (Hue *et al*, 2005). The importance of ERK1/2 activation in melanoma progression has also been reported (Zhuang *et al*, 2005).

In conclusion, our results suggest that CXCR1 and CXCR2 play important roles in the regulation of human melanoma tumourigenesis and progression. Ectopic expression of CXCR1 or CXCR2 confers a more aggressive phenotype to melanoma cells. Moreover, CXCR1 and CXCR2 are implicated in tumour growth by directly enhancing tumour cell properties, thus indicating the possibility of manipulating CXCR1 and CXCR2 for future therapeutic intervention.
