# Supplementary figures and images for: CXCR1 and CXCR2 enhances human melanoma tumourigenesis, growth and invasion
Source: Br J Cancer. 2009 Apr 28;100(10):1638–46. doi: 10.1038/sj.bjc.6605055 (PMC2696769; doi:10.1038/sj.bjc.6605055)

## Slide 1
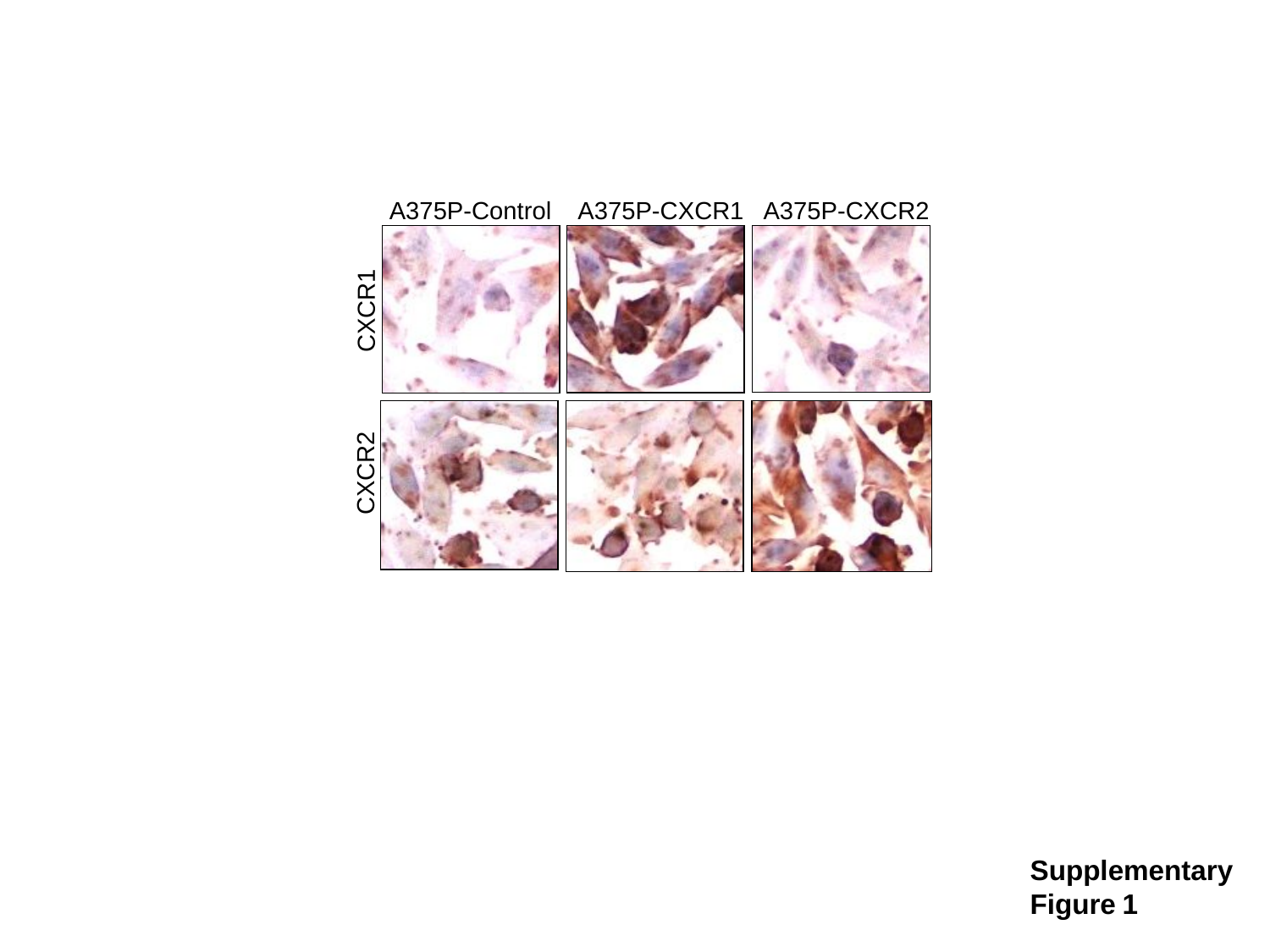

A375P-Control A375P-CXCR1 A375P-CXCR2
CXCR1
CXCR2
Supplementary
Figure 1

Supplement: Supplementary Figure 1 [file 6605055x1.ppt]

## Slide 1
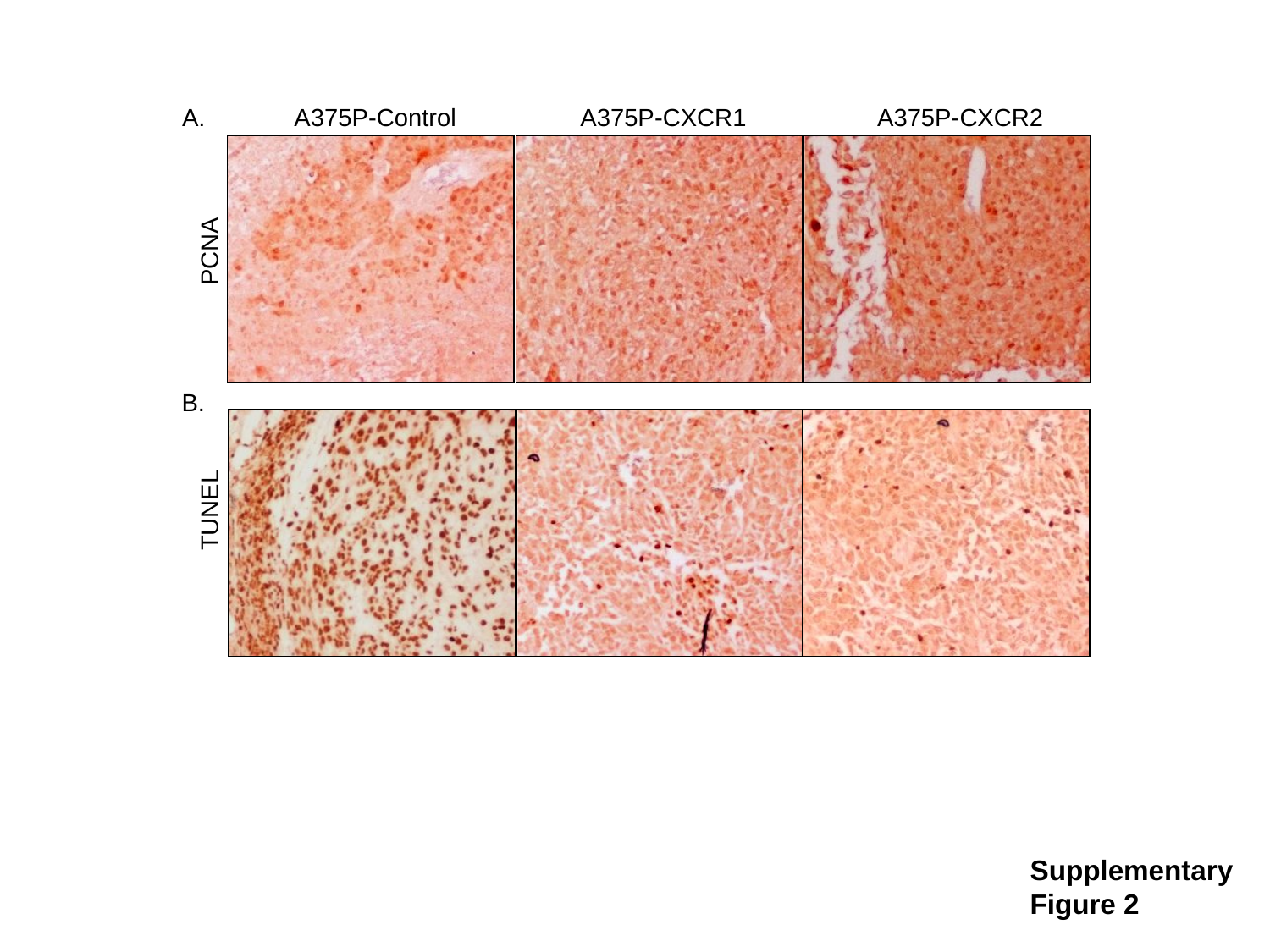

A.
A375P-Control A375P-CXCR1 A375P-CXCR2
PCNA
B.
TUNEL
Supplementary
Figure 2

Supplement: Supplementary Figure 2 [file 6605055x2.ppt]
