# Supplementary material for: CXCR1 and CXCR2 enhances human melanoma tumourigenesis, growth and invasion
Source: Br J Cancer. 2009 Apr 28;100(10):1638–46. doi: 10.1038/sj.bjc.6605055 (PMC2696769; doi:10.1038/sj.bjc.6605055)
Supplement: Supplementary Figure Legends [file 6605055x3.doc]

**Supplementary Figure 1. Immunocytochemical staining for CXCR1 and CXCR2 expression in A375P melanoma cells.**

Immunocytochemical staining for CXCR1 and CXCR2 were analyzed based on DAB staining as described in Material and Methods.

**Supplementary Figure 2. PCNA and TUNEL immunohistochemical staining of tumors.** Immunohistochemical staining for PCNA and TUNEL were analyzed based on DAB staining as described in Material and Methods. In A375P-group tumors showed an imbalance of PCNA to TUNEL positive cells.
